# Supplementary material for: Activation of TrkB signaling mitigates cerebellar anomalies caused by Rbm4-Bdnf deficiency
Source: Commun Biol. 2023 Sep 5;6:910. doi: 10.1038/s42003-023-05294-z (PMC10480162; doi:10.1038/s42003-023-05294-z)
Supplement: Supplementary file 2 — Supplementary information [file 42003_2023_5294_MOESM2_ESM.pdf]

Supplementary Information

Activation of TrkB signaling mitigates cerebellar anomalies caused by *Rbm4-Bdnf* deficiency

Yu-Young Tsai[1], Chiu-Lun Shen[1,2], Dhananjaya D[1], Ching-Yen Tsai[3], and Woan-Yuh Tarn[1,*]

[1]Institute of Biomedical Sciences, Academia Sinica, Taipei, Taiwan
[2]Institute of Molecular Medicine, College of Medicine, National Taiwan University, Taipei, Taiwan
[3]Institute of Molecular Biology, Academia Sinica, Taipei, Taiwan

*Corresponding author
Woan-Yuh Tarn, e-mail: wtarn@ibms.sinica.edu.tw; ORCID: 0000-0001-6244-6639

This Supplementary Information PDF contains the following:

Supplementary Figures

- Supplementary Fig. 1. General characterization of *Rbm4*dKO mice.
- Supplementary Fig. 2. Rotarod analysis of *Rbm4*dKO mice.
- Supplementary Fig. 3. General characterization of GCs and PCs in the developing *Rbm4*dKO cerebellum.
- Supplementary Fig. 4. Prenatal treatment with 7,8-DHF promotes cerebellar development of *Rbm4*dKO pups.
- Supplementary Fig. 5. Prenatal supplementation with 7,8-DHF improves the rotarod performance of *Rbm4*dKO pups.
- Supplementary Fig. 6. RBM4 controls BDNF expression via alternative splicing of *Bdnf* transactivators.
- Supplementary Fig. 7. Original scanned images of the blots and gels presented in this study.

Supplementary Table

Supplementary Table 1. Primers used in this study.

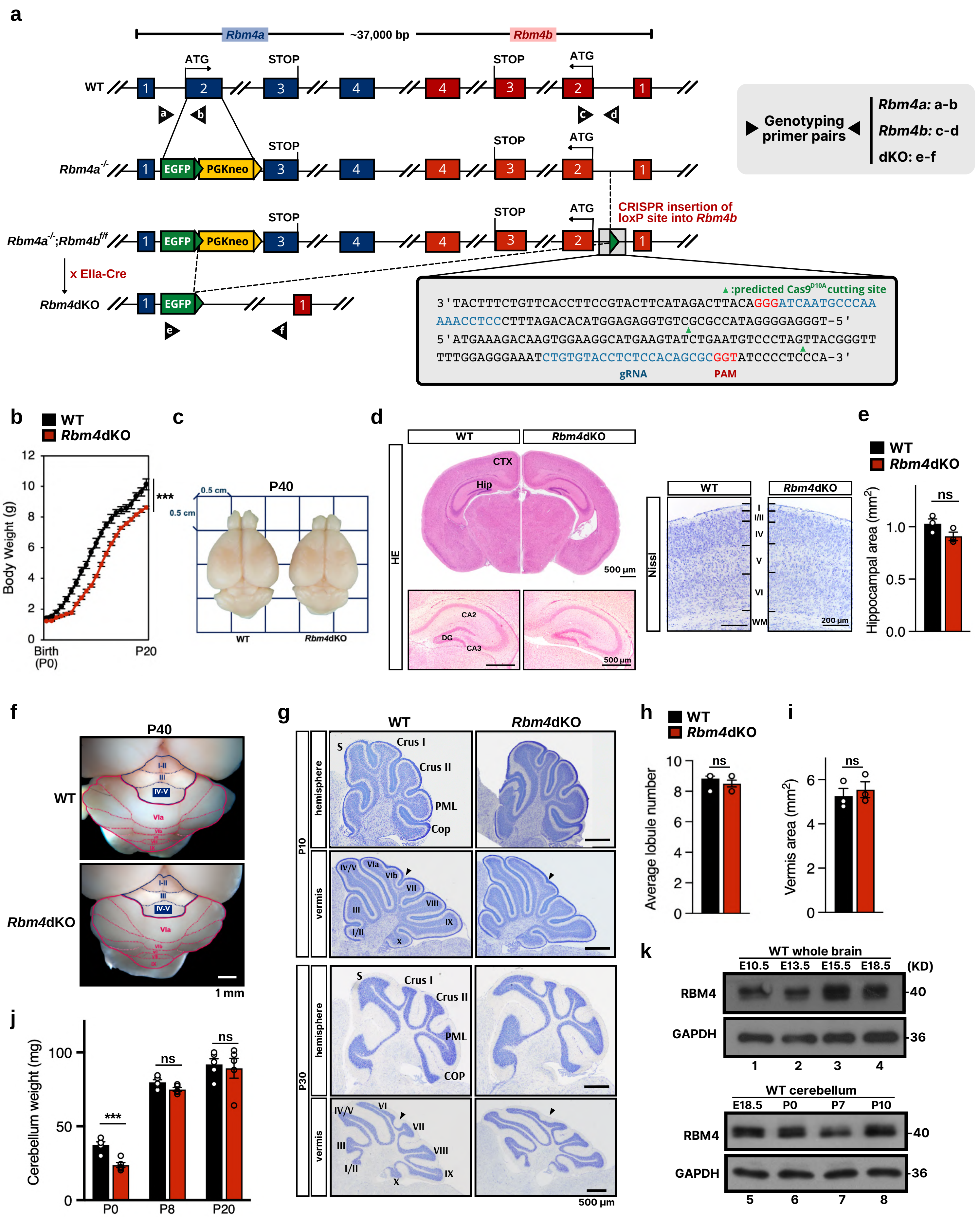

a
Rbm4a
~37,000 bp
Rbm4b
WT
ATG
STOP
STOP
ATG
Rbm4a-/-
EGFP
PGKneo
Rbm4a-/-;Rbm4bf/f
CRISPR insertion of loxP site into Rbm4b
x Ella-Cre
Rbm4dKO
Genotyping primer pairs
Rbm4a: a-b
Rbm4b: c-d
dKO: e-f
▲:predicted Cas9D10A cutting site
3'TACTTTCTGTTCACCTTCCGTACTTCATAGACTTACAGGGATCAATGCCCAA
AAACCTCCCTTTAGACACATGGAGAGGTGTCGCGCCATAGGGGAGGGT-5'
5'ATGAAAGACAAGTGGAAGGCATGAAGTATCTGAATGTCCCTAGTTACGGGTT
TTTGGAGGGAAATCTGTGTACCTCTCCACAGCGCGGTATCCCCTCCCA-3'
gRNA
PAM
b
WT
Rbm4dKO
Body Weight (g)
***
Birth (P0)
P20
c
P40
0.5 cm
WT
Rbm4dKO
d
HE
CTX
Hip
500 μm
CA2
DG
CA3
Nissl
I
I/II
IV
V
VI
WM
200 μm
e
Hippocampal area (mm2)
ns
f
P40
I-II
III
IV-V
VIa
VIb
VII
VIII
IX
1 mm
g
WT
Rbm4dKO
P10
P30
hemisphere
vermis
S
Crus I
Crus II
PML
Cop
COP
IV/V
VIa
VIb
VI
VII
VIII
IX
X
III
I/II
500 μm
h
Average lobule number
ns
i
Vermis area (mm2)
ns
j
Cerebellum weight (mg)
***
ns
ns
P0
P8
P20
k
WT whole brain
E10.5
E13.5
E15.5
E18.5
(KD)
RBM4
GAPDH
-40
-36
1
2
3
4
WT cerebellum
E18.5
P0
P7
P10
5
6
7
8

Supplementary Fig. 1 General characterization of *Rbm4*dKO mice.

a) Detailed scheme of the *Rbm4a/b* knockout strategy. Single *Rbm4* knockout mice had been generated previously[6]. A loxP site was inserted into intron 1 of *Rbm4b* using CRISPR-Cas9A^{D10A}-mediated gene editing (grey box). *Rbm4*dKO (*Rbm4a*$^{-/-}$;*Rbm4b*$^{-/-}$) mice were obtained by mating *Rbm4a*$^{-/-}$;*Rbm4*$^{f/f}$ with *EIIa-Cre* mice. The primers for genotyping (Fig. 1b) were pair-confined as indicated by the black arrowheads (a–f).
b) Body weight of WT and *Rbm4*dKO mice from birth to P20 (N=7 WT; N=15 *Rbm4*dKO).
c) Representative images of the whole brain of WT and *Rbm4*dKO at P10.
d) Representative coronal sections of the cortex stained with HE (upper left panel), magnified hippocampal structure (lower left panel), and enlarged cortical layers stained with cresyl violet (Nissl; right panel) at P10 of each genotype. Roman numerals indicate the six cortical layers.
e) Average hippocampal area of P30 WT and *Rbm4*dKO (N=3 per group).
f) Representative images of the posterior view of WT and *Rbm4*dKO cerebellum marked with lobulation at P40.
g) Nissl-stained sagittal sections of the cerebellar vermis and hemisphere of WT and *Rbm4*dKO mice at P10 and P30.
h) Average number of lobules in P30 WT and *Rbm4*dKO (N=6 per group).
i) Vermal area of P30 WT and *Rbm4*dKO mice (N=3 per group).
j) Bar graph shows the average weight of the cerebellum at the indicated ages (N=5 per genotype).
k) Immunoblotting of RBM4 in the wild-type embryonic brain (lanes 1–4) and cerebellum (lanes 5–8) at the indicated developmental days. GAPDH was used as the control. Arrowheads indicate the presence or absence of an icf.

Roman numerals indicate relevant lobules of the vermis.

Abbreviations: CTX, cortex; Hip, hippocampus; DG, dentate gyrus; CA, cornu ammonis; WM, white matter; S, simple lobule; PML, paramedian lobe; COP, copula pyramidis. *P*-values were determined with the Student's *t* test: $^{*}P < 0.05$, $^{**}P < 0.01$, $^{***}P < 0.001$; ns, difference not statistically significant. Error bars represent standard error of the mean.

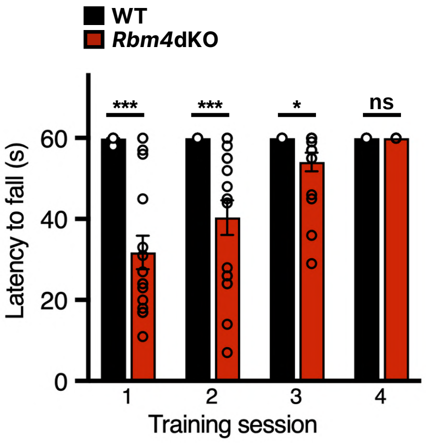

Supplementary Fig. 2 Rotarod analysis of *Rbm4*dKO mice. Rotarod analysis was performed as in Fig. 2a. Bar graph shows the average latency to fall (s) during the low-constant-speed (0, 4, 4, 4 rpm in respective session 1, 2, 3, 4) training sessions (N=17 per genotype).

P-values and error bars are the same as in Supplementary Fig. 1.

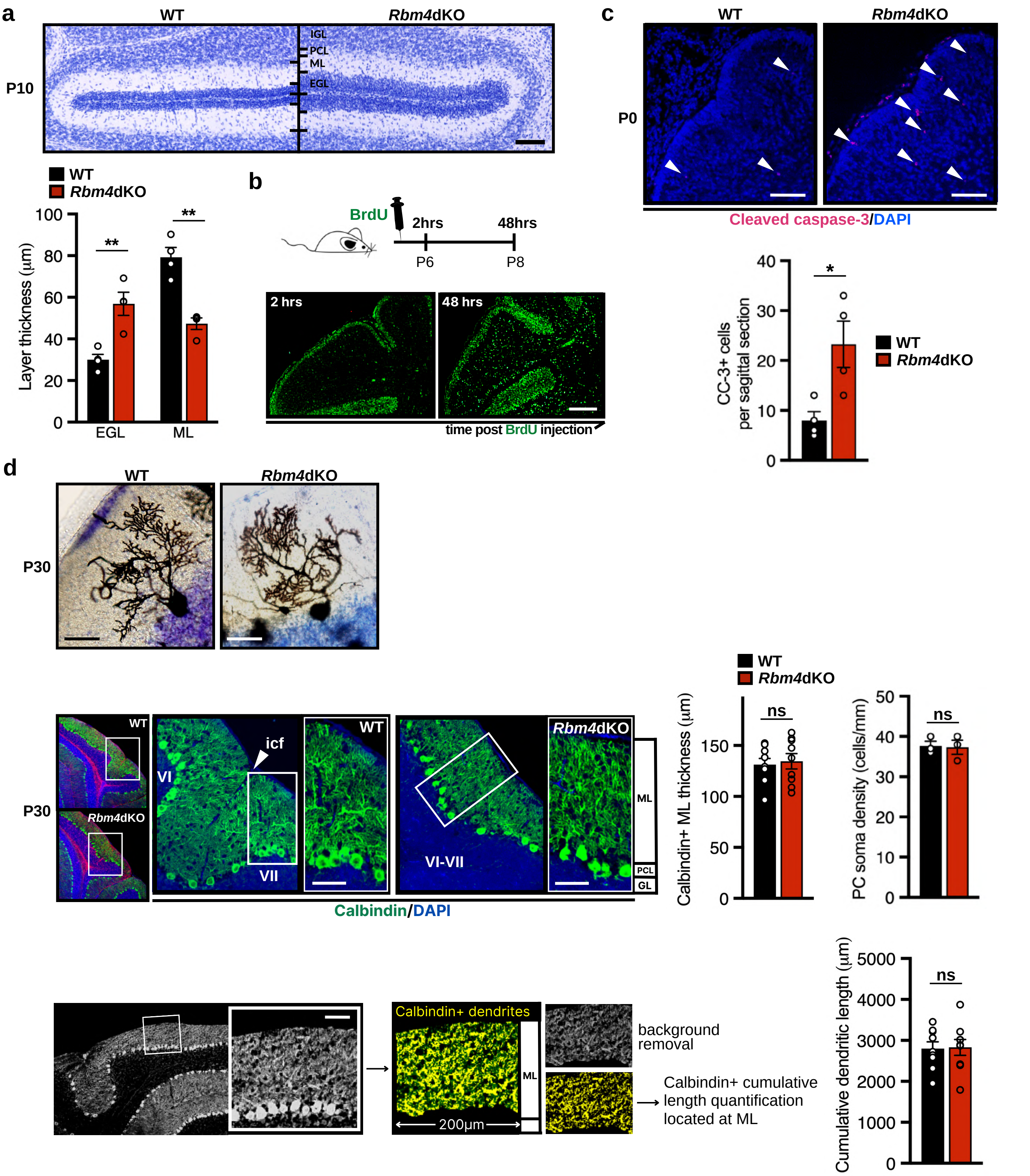

Supplementary Fig. 3 General characterization of GCs and PCs in the developing *Rbm4*dKO cerebellum.

a) Hematoxylin-stained sagittal vermis of the P10 cerebellum. Scale bar, 100 μm. Bar graph (bottom) shows the average thickness of the EGL and molecular layer (ML) at P10 (N=4).

b) Schematic of the BrdU pulse-labeling strategy. BrdU was intraperitoneally injected into WT and *Rbm4*dKO mice at P6 (50 mg/kg), and the brain was harvested either 2 or 48 h post-injection. Immunofluorescence staining for BrdU was performed on sagittal sections of the cerebellum, as shown at the bottom.

c) Immunofluorescence staining against cleaved caspase-3 (CC-3) in the WT and *Rbm4*dKO cerebellum (N=4 per group).

d) PC morphology at P30 via Golgi staining (upper panels) and immunofluorescence against calbindin (middle panels). PC dendritic complexity was measured with ML thickness, soma density (N=3 per genotype), and cumulative dendritic length per field of 200 µm by width (randomly selected region of interest for quantification; N=9 fields per genotype). Cumulative dendritic length was quantified from MetaMorph software by applying fluorescence mask (yellow; bottom panels) to minimize staining background; fluorescence signal that met intensity threshold was measured.

Scale bars, 100 µm (panels a-c); 50 µm (panel d). *P*-values and error bars are the same as in Supplementary Fig. 1.

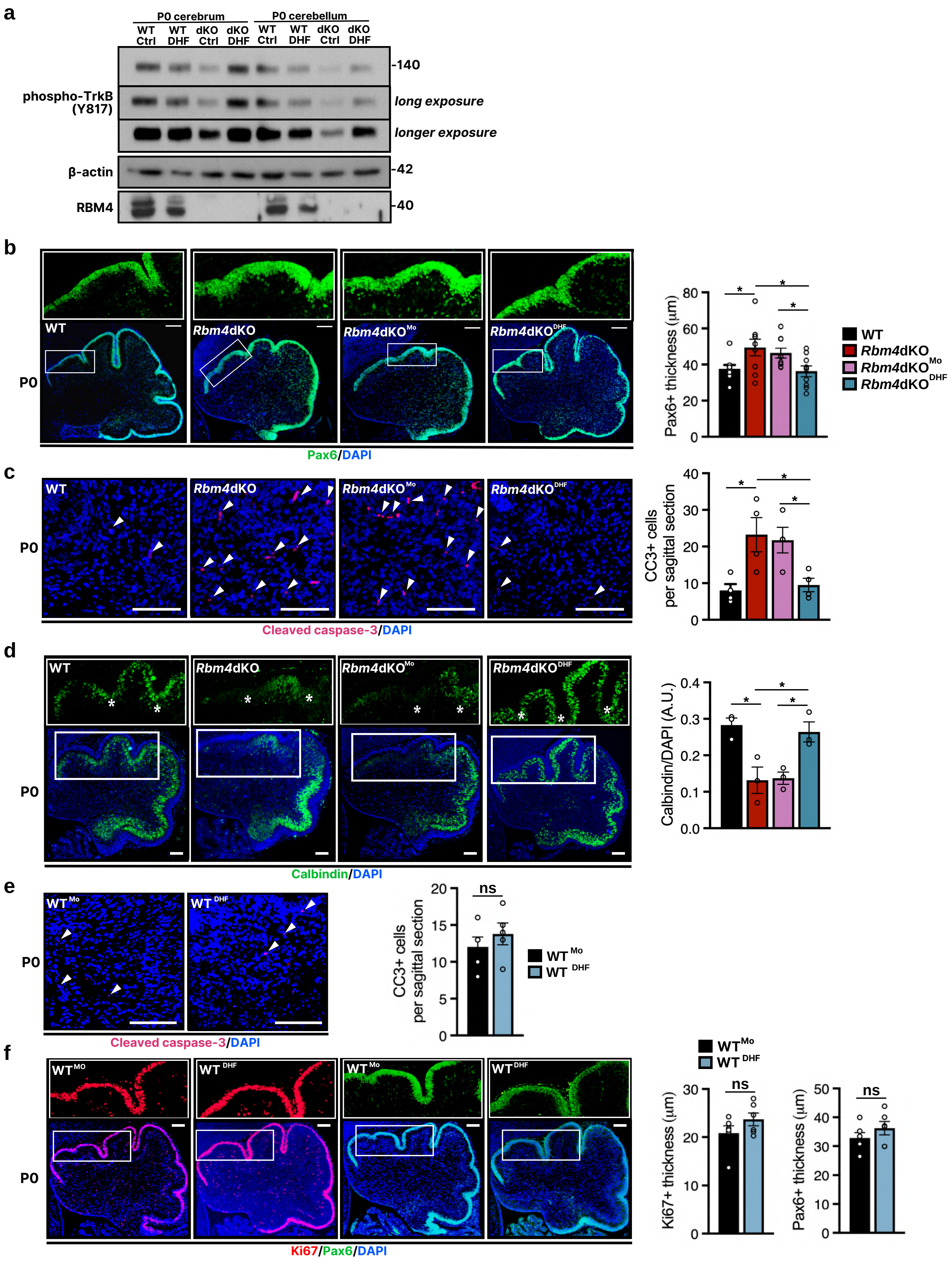

a
P0 cerebrum
P0 cerebellum
WT Ctrl
WT DHF
dKO Ctrl
dKO DHF
phospho-TrkB (Y817)
-140
long exposure
longer exposure
β-actin
-42
RBM4
-40
b
WT
Rbm4dKO
Rbm4dKO^Mo
Rbm4dKO^DHF
P0
Pax6/DAPI
Pax6+ thickness (μm)
c
Cleaved caspase-3/DAPI
CC3+ cells per sagittal section
d
Calbindin/DAPI
Calbindin/DAPI (A.U.)
e
WT^Mo
WT^DHF
ns
f
Ki67/Pax6/DAPI
Ki67+ thickness (μm)

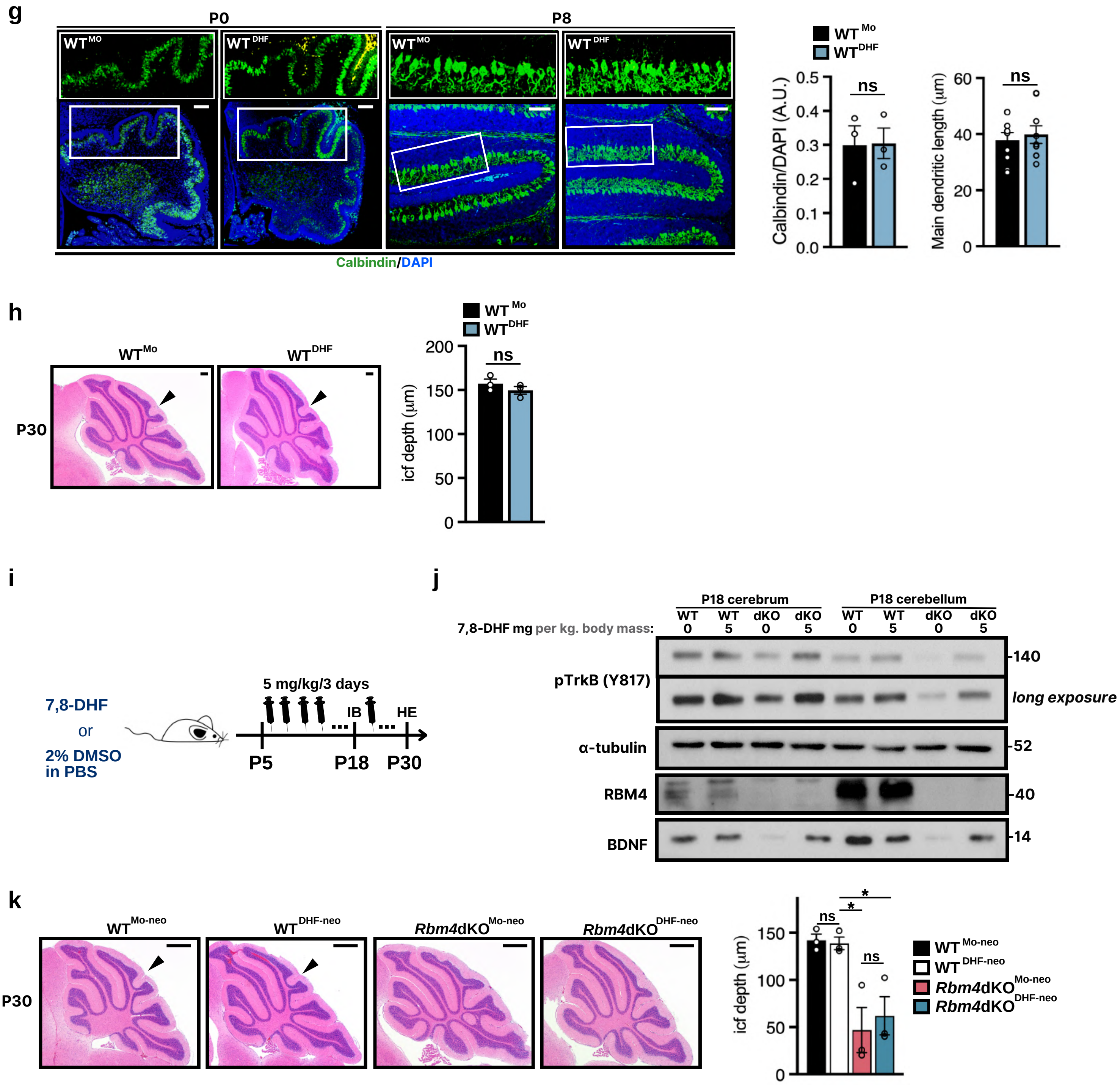

Supplementary Fig. 4 Prenatal treatment with 7,8-DHF promotes cerebellar development of *Rbm4*dKO pups.

a) Immunoblotting profile of phospho-TrkB (Y817) in cerebrum and cerebellum of newborn pups that were prenatally supplemented with 7,8-DHF.

b) Representative Pax6 immunofluorescence staining of the P0 cerebellum from the indicated genotype/treatment. Regions marked by white boxes are magnified in the lower panels. Bar graph shows the average Pax6+ thickness in the P0 WT, *Rbm4*dKO, *Rbm4*dKO^{Mo}, and *Rbm4*dKO^{DHF} (N=3 per group).

c) Immunostaining and quantification of apoptotic cells in P0 WT, *Rbm4*dKO, *Rbm4*dKO^{Mo}, and *Rbm4*dKO^{DHF} (N=4 per group).

d) Representative immunofluorescence staining for calbindin and average fluorescence intensity (normalized to DAPI) quantified in P0 WT, *Rbm4*dKO, *Rbm4*dKO^{Mo}, and *Rbm4*dKO^{DHF} (N=3 per group). The anterior region of the vermis is magnified and shown above. Asterisks mark the anchoring center of the fissures.

e) Immunostaining against CC3+ cells in P0 WT^{Mo} and WT^{DHF} (N=3 per group).

f) Representative Ki67 and Pax6 immunofluorescence staining of the P0 cerebellum from the indicated genotype/ treatment. Regions marked by white boxes are magnified in the upper panels. Bar graph respectively shows the average Ki67+ and Pax6+ thickness in the P0 WT^{Mo} and WT^{DHF} (N=6 per group).

g) Representative immunofluorescence staining for calbindin. The average fluorescence intensity (normalized to DAPI) was quantified in P0 WT^{Mo} and WT^{DHF} (N=3 per group), and the main dendritic length was measured in P8 littermates (N=8 per group).
h) Representative HE staining of WT^{Mo} and WT^{DHF} cerebellar vermis is shown at P30. Bar graph shows average icf depth.
i) Treatment schematic of neonatal injection, generating WT/*Rbm4*$dKO^{Mo\text{-}neo}$ and WT/*Rbm4*$dKO^{DHF\text{-}neo}$.
j) Immunoblotting profile of the neonatally treated groups.
k) HE staining of cerebellar vermis at P30 from the neonatal injection groups. Bar graph (right) shows average of icf depth (N=3 per genotype).

Abbreviation: A.U., arbitrary unit.
Scale bars, 100μm (panels b-g); 200μm (panel h); 500μm (panel k). *P*-values and error bars are the same as in Supplementary Fig. 1.

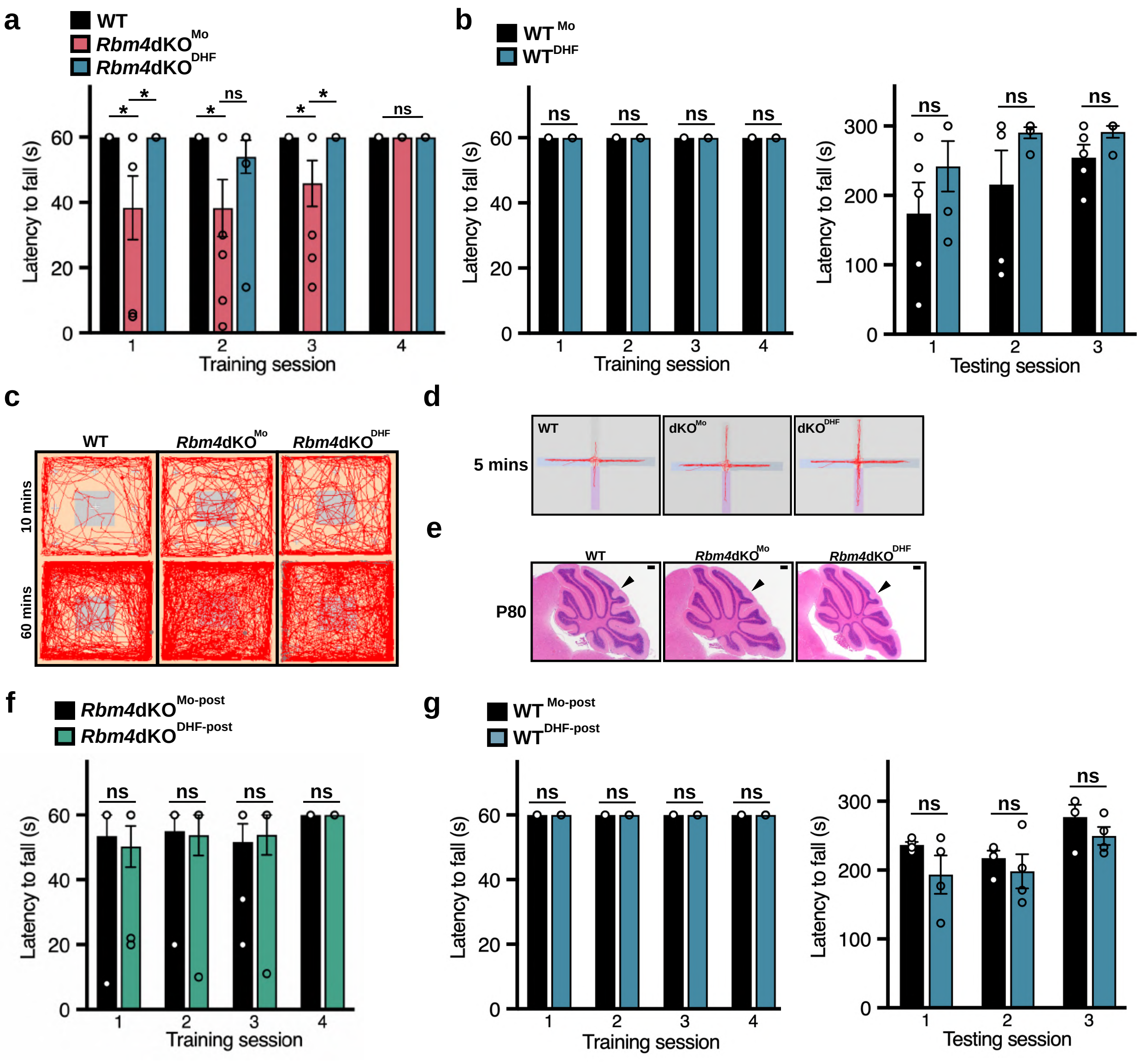

Supplementary Fig. 5 Prenatal supplementation with 7,8-DHF improves the rotarod performance of *Rbm4*dKO pups.

a) Bar graph shows the rotarod performance of each group during the training trials. Sample size was the same as in Fig. 7a.
b) Rotarod analysis was analogously performed in prenatally treated WT animals (N=5 per group).
c) Representative mouse movement tracks during the open-field test over the habituation period (10 and 60 mins). The number of mice subjected to behavioral analyses was the same as in Fig. 7b.
d) Representative mouse movement tracks during the elevated plus maze test over the 5-min habituation period. The number of mice subjected to behavioral analyses was the same as in Fig. 7c.
e) Representative HE staining of the P80 sagittal vermis. Arrowheads indicate the presence or absence of an icf.
f) The training sessions of rotarod analysis in chronically treated *Rbm4*dKO are shown (N=8 per group).
g) Rotarod analysis was analogously performed in chronically treated WT mice (N=4 per group).

Scale bars, 200µm. *P*-values and error bars are the same as in Supplementary Fig. 1.

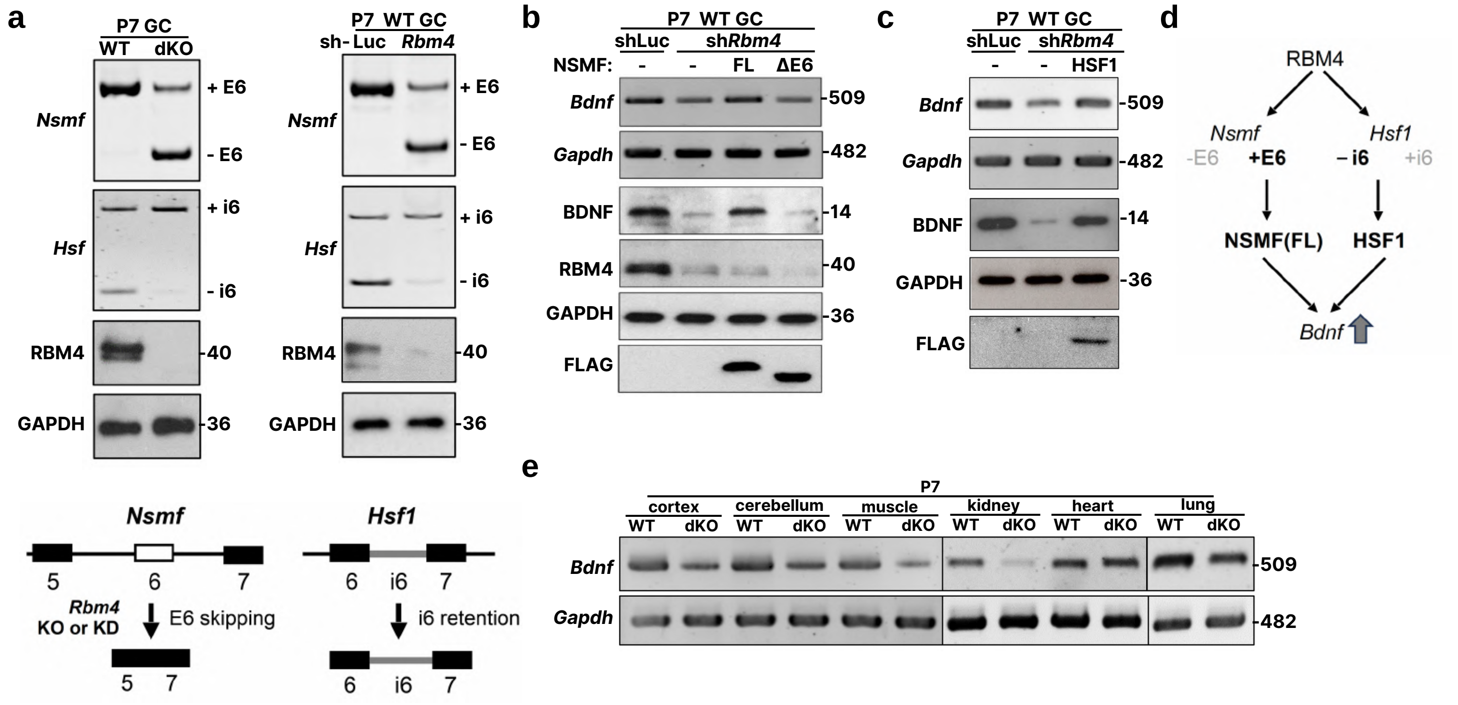

Supplementary Fig. 6 RBM4 controls BDNF expression via alternative splicing of *Bdnf* transactivators.

a) RT-PCR of *Nsmf* and *Hsf1*, and immunoblotting of RBM4 and GAPDH in P7 WT and *Rbm4*dKO GCs (left panel) and shRNA (Luc) or sh*RBM4*-transfected WT GCs (right panel). Diagram shows that *Rbm4* knockout (KO) or knockdown (KD) induces exon 6 skipping of *Nsmf* and intron 6 retention of *Hsf1*.

b) RT-PCR and immunoblotting in WT GCs that were transfected with control shRNA (Luc), or *Rbm4* shRNA together with the empty vector (-) or vector expressing FLAG-tagged NSMF (FL and ΔE6).

c) RT-PCR and immunoblotting in WT GCs that were transfected with control shRNA (Luc) or *Rbm4* shRNA together with the empty (-) or FLAG-HSF1 expression vector.

d) Diagram shows the model: RBM4 regulates splicing of *Nsmf* and *Hsf1*, thereby increasing the expression of full-length NSMF (exon 6 inclusion) and HSF1. Both NSMF and HSF1 can transactivate *Bdnf.*

e) RT-PCR of *Bdnf* and *Gapdh* (control) was performed in the indicated tissues of P7 WT and *Rbm4*dKO mice.

Figure 1b

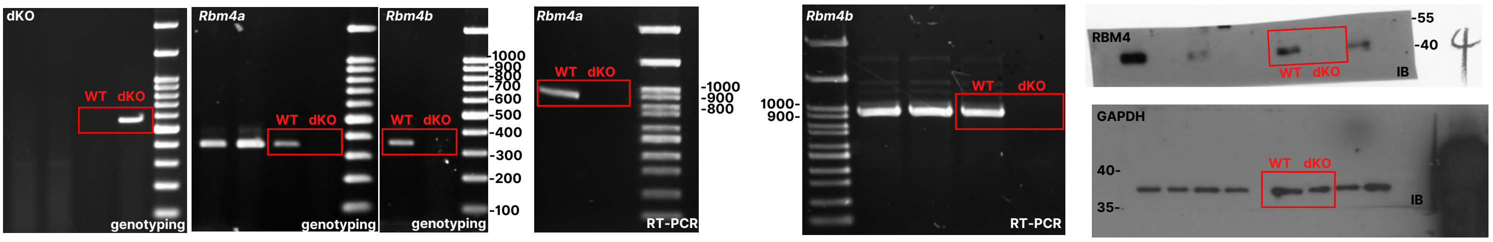

Figure 4a

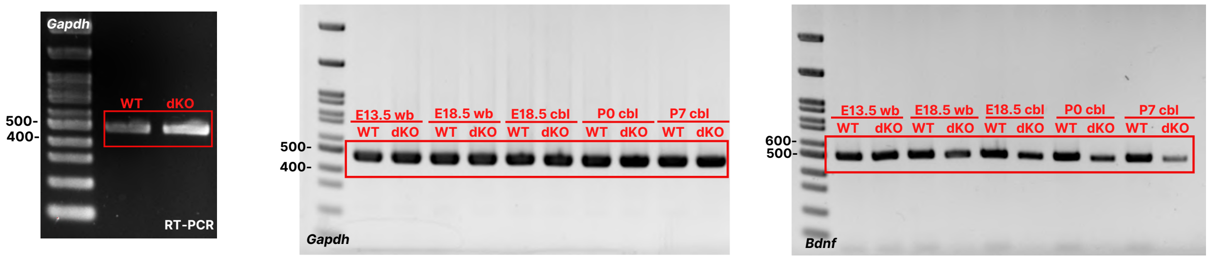

Figure 4b

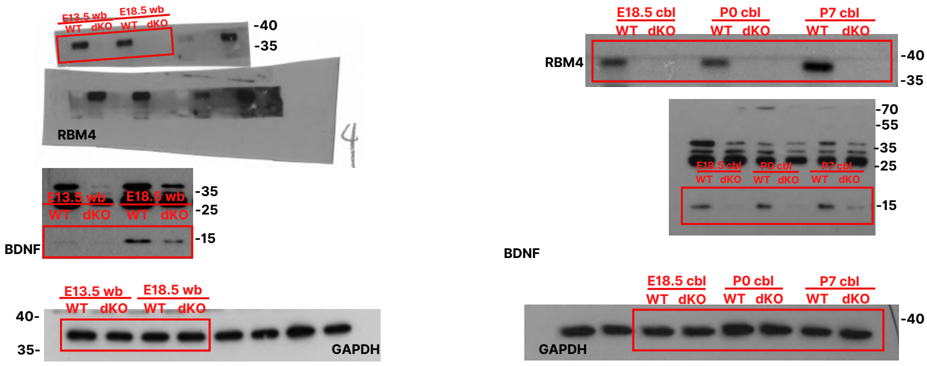

Figure 4c

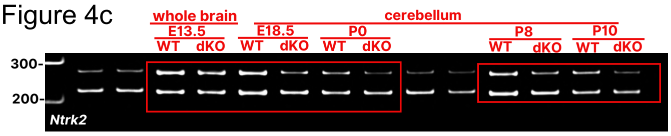

Figure 4d

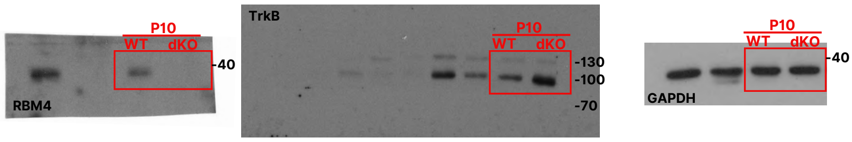

Figure 5b

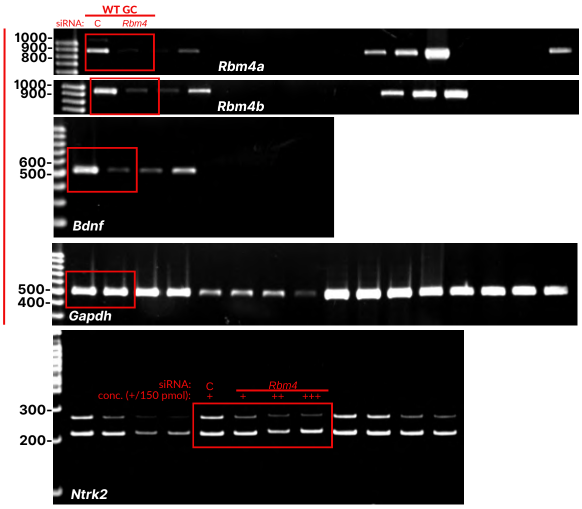

Figure 5c

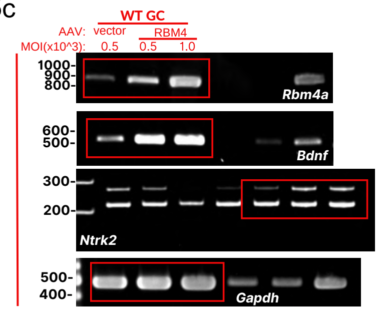

Figure 5d

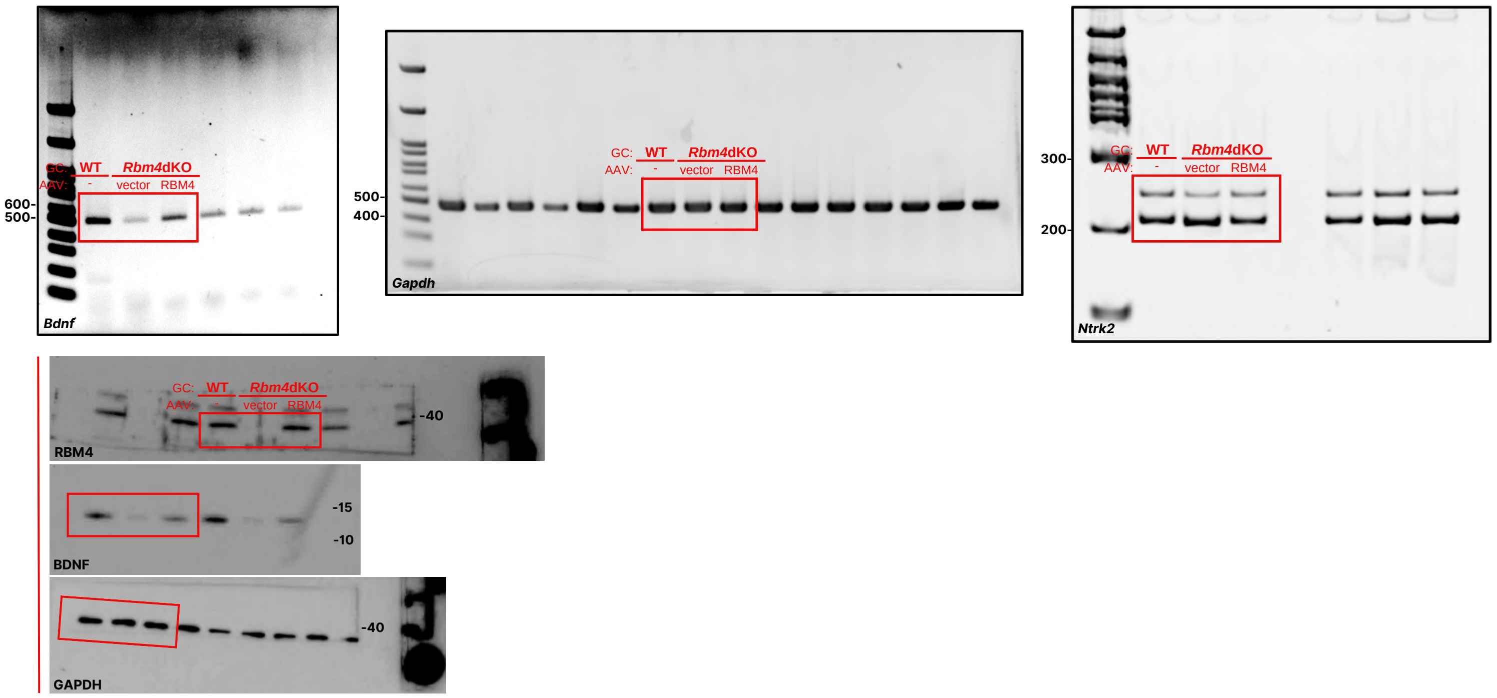

Figure 6b

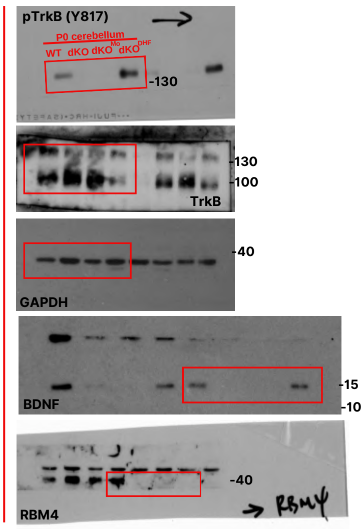

Figure 7f

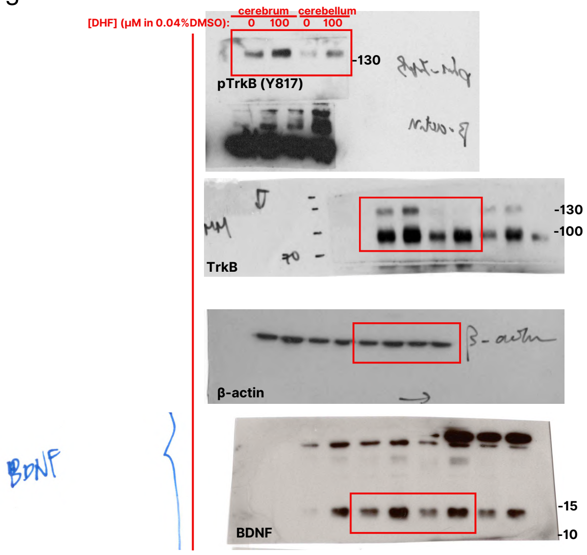

Supplementary Figure 1k

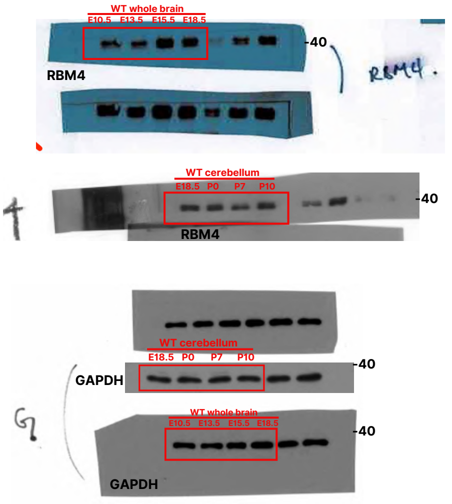

Supplementary Figure 4a

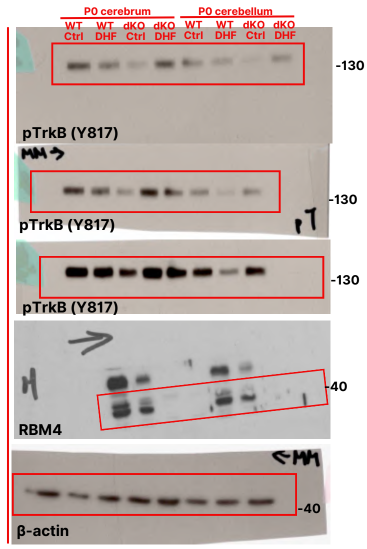

Supplementary Figure 4j

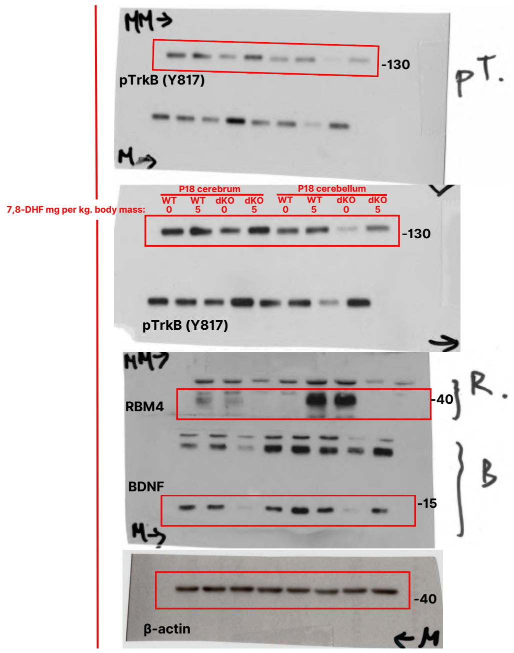

Supplementary Figure 6a

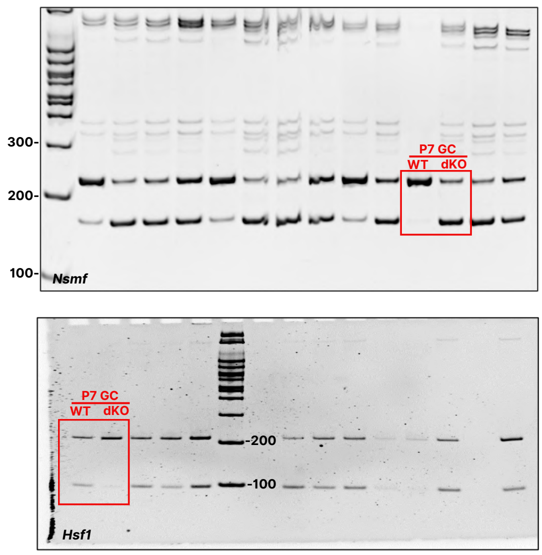

Supplementary Figure 6a- continued.

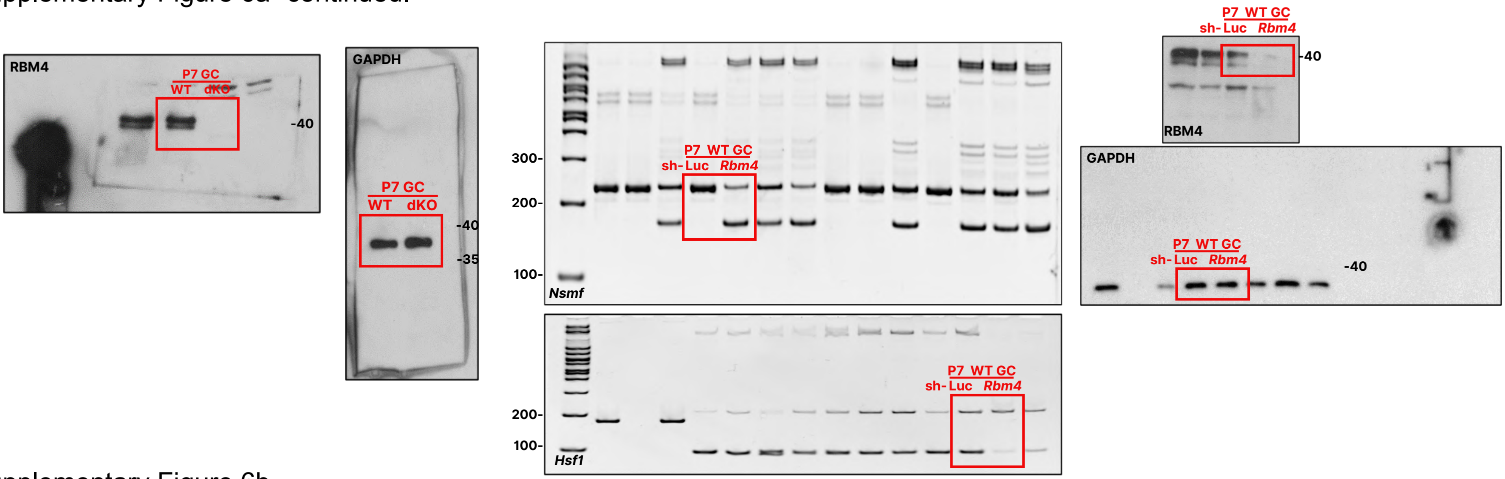

Supplementary Figure 6b

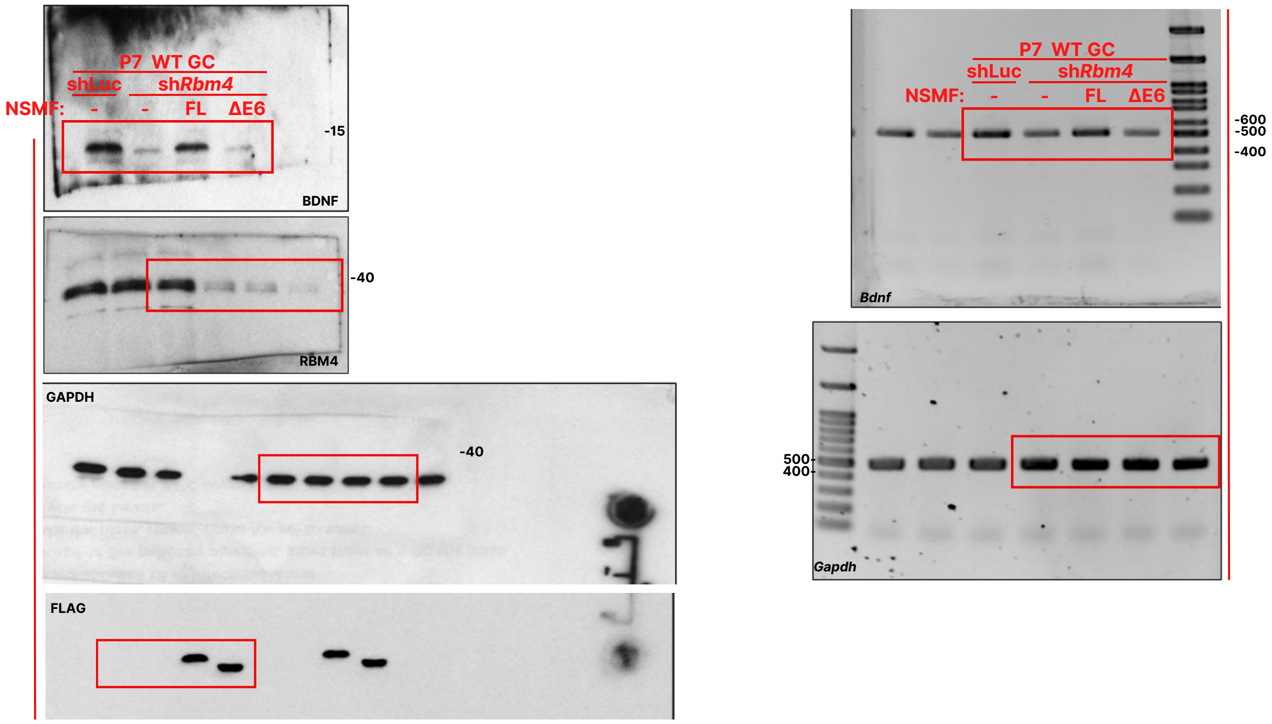

Supplementary Figure 6c

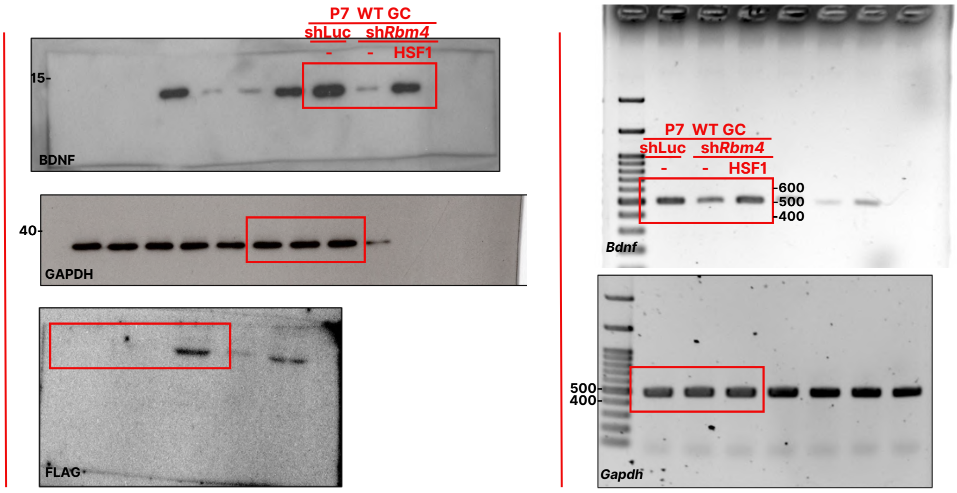

Supplementary Figure 6e

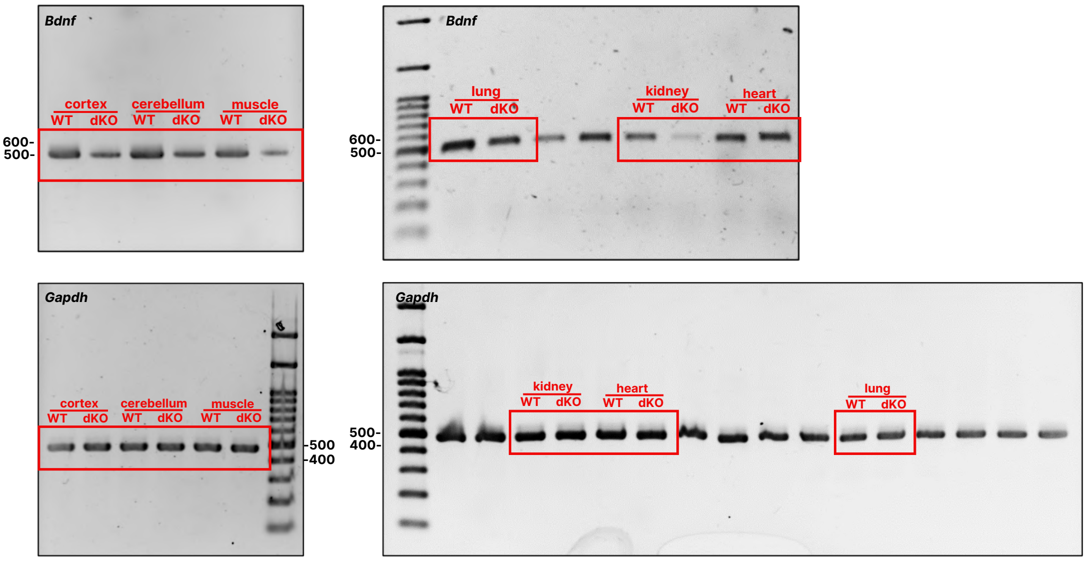

Supplementary Fig. 7 Original scanned images of the blots and gels presented in this study. The figure panel that corresponds to each scan is labeled, and the selected region for display is marked in red box with sample labels.

Supplementary Table 1 Primers used in this study.

Gene name	5'-Oligonucleotide sequence-3'	Usage
Rbm4a	Forward: TTCTTGTGAGAAATAGAAGC<br>Reverse: GCACAAAGCCATAATTCTTA	Genotyping
Rbm4b	Forward: CGTCTTGTCCTCTATGTGC<br>Reverse: GCTAACCTTGTAGAGTCAATGTGC	Genotyping
*Rbm4*dKO	Forward: GCGGCCGCGACTCTAGATC<br>Reverse: TTTGGGGCCACTCATCTTCT	Genotyping
Rbm4a	Forward: ATGGTGAAGCTGTTCATTGG<br>Reverse: CTGCTGCTGCTGCAGCTCCTGAAGG	RT-PCR
Rbm4b	Forward: ATGGTGAAGCTGTTCATTGG<br>Reverse: CGGGAAGCACAGCCGCATTC	RT-PCR
Gapdh	Forward: GTCGTGGAGTCTACTGGTGT<br>Reverse: TACTTGGCAGGTTTCTCCAG	RT-(q)PCR
Bdnf	Forward: TGGCTGACACTTTTGAGCAC<br>Reverse: CCAGCCAATTCTCTTTTTGC	RT-(q)PCR
Ntrk2 fl	Forward: GCCAACTGACATTGGGGATA<br>Reverse: CCTCCGAAGAAGATGGAGTG	RT-PCR
Ntrk2 t1	Forward: GCCAACTGACATTGGGGATA<br>Reverse: TACCCATCCAGTGGGATCTT	RT-PCR
Nsmf E6	Forward: ACCTTTTCAGCTTCCAGACG<br>Reverse: GGGAAGTGTCGGCTTTCATA	RT-PCR
Hsf1 i6	Forward: TCTCACTGGTGCAGTCGAAC<br>Reverse: CGACCATACTTGGGCACAG	RT-PCR
Nsmf (ΔE6)	Forward: GAACTTCCGCAAACACCTG<br>Reverse: GAGATGGCTTGCATAGTTG	plasmid construction<br>mutagenesis

Species: mouse.
