# Supplementary material for: Activation of TrkB signaling mitigates cerebellar anomalies caused by Rbm4-Bdnf deficiency
Source: Commun Biol. 2023 Sep 5;6:910. doi: 10.1038/s42003-023-05294-z (PMC10480162; doi:10.1038/s42003-023-05294-z)
Supplement: Supplementary file 3 — Description of Additional Supplementary Files [file 42003_2023_5294_MOESM3_ESM.pdf]

Description of Additional Supplementary Files

File name: Supplementary Data

Description: The Excel file provided contains the numerical source data underlying the graphs presented in this study.
