# Supplementary material for: Activation of TrkB signaling mitigates cerebellar anomalies caused by Rbm4-Bdnf deficiency
Source: Commun Biol. 2023 Sep 5;6:910. doi: 10.1038/s42003-023-05294-z (PMC10480162; doi:10.1038/s42003-023-05294-z)
Supplement: Supplementary file 5 — Reporting Summary [file 42003_2023_5294_MOESM5_ESM.pdf]

nature portfolio

Corresponding author(s): Dr. Woan-Yuh Tarn

Last updated by author(s): Aug 21, 2023

Reporting Summary

Nature Portfolio wishes to improve the reproducibility of the work that we publish. This form provides structure for consistency and transparency in reporting. For further information on Nature Portfolio policies, see our Editorial Policies and the Editorial Policy Checklist.

Statistics

For all statistical analyses, confirm that the following items are present in the figure legend, table legend, main text, or Methods section.

n/a	Confirmed	
☐	☒	The exact sample size (*n*) for each experimental group/condition, given as a discrete number and unit of measurement
☐	☒	A statement on whether measurements were taken from distinct samples or whether the same sample was measured repeatedly
☐	☒	The statistical test(s) used AND whether they are one- or two-sided<br>*Only common tests should be described solely by name; describe more complex techniques in the Methods section.*
☒	☐	A description of all covariates tested
☐	☒	A description of any assumptions or corrections, such as tests of normality and adjustment for multiple comparisons
☐	☒	A full description of the statistical parameters including central tendency (e.g. means) or other basic estimates (e.g. regression coefficient) AND variation (e.g. standard deviation) or associated estimates of uncertainty (e.g. confidence intervals)
☐	☒	For null hypothesis testing, the test statistic (e.g. *F*, *t*, *r*) with confidence intervals, effect sizes, degrees of freedom and *P* value noted<br>*Give P values as exact values whenever suitable.*
☒	☐	For Bayesian analysis, information on the choice of priors and Markov chain Monte Carlo settings
☒	☐	For hierarchical and complex designs, identification of the appropriate level for tests and full reporting of outcomes
☒	☐	Estimates of effect sizes (e.g. Cohen's *d*, Pearson's *r*), indicating how they were calculated

Our web collection on statistics for biologists contains articles on many of the points above.

Software and code

Policy information about availability of computer code

Data collection	No custom code nor open source software was used for data collection.
Data analysis	RNA-sequencing data analysis: Shen S., Park JW., Lu ZX., Lin L., Henry MD., Wu YN., Zhou Q., Xing Y. (2014) rMATS: Robust and Flexible Detection of Differential Alternative Splicing from Replicate RNA-Seq Data. PNAS, 111(51):E5593-601.

For manuscripts utilizing custom algorithms or software that are central to the research but not yet described in published literature, software must be made available to editors and reviewers. We strongly encourage code deposition in a community repository (e.g. GitHub). See the Nature Portfolio guidelines for submitting code & software for further information.

Data

Policy information about availability of data

All manuscripts must include a data availability statement. This statement should provide the following information, where applicable:

- Accession codes, unique identifiers, or web links for publicly available datasets
- A description of any restrictions on data availability
- For clinical datasets or third party data, please ensure that the statement adheres to our policy

A data availability statement has been included in the main article.
Other data generated during and/or analysed for the current study are available from the corresponding author upon reasonable request.

Human research participants

Policy information about studies involving human research participants and Sex and Gender in Research.

Reporting on sex and gender	N/A
Population characteristics	N/A
Recruitment	N/A
Ethics oversight	N/A

Note that full information on the approval of the study protocol must also be provided in the manuscript.

Field-specific reporting

Please select the one below that is the best fit for your research. If you are not sure, read the appropriate sections before making your selection.

☒ Life sciences ☐ Behavioural & social sciences ☐ Ecological, evolutionary & environmental sciences

For a reference copy of the document with all sections, see nature.com/documents/nr-reporting-summary-flat.pdf

Life sciences study design

All studies must disclose on these points even when the disclosure is negative.

Sample size	No statistical method was used to pre-determine sample size.<br><br>Sample sizes used to determine statistical significance were based on similar publications:<br>1. Kawamura A, Katayama Y, Kakegawa W, et al. The autism-associated protein CHD8 is required for cerebellar development and motor function. Cell Rep. 2021;35(1):108932. doi:10.1016/j.celrep.2021.108932<br>2. Yang H, Zhu Q, Cheng J, et al. Opposite regulation of Wnt/β-catenin and Shh signaling pathways by Rack1 controls mammalian cerebellar development. Proc Natl Acad Sci U S A. 2019;116(10):4661-4670. doi:10.1073/pnas.1813244116<br>3. Ryan KE, Kim PS, Fleming JT, et al. Lkb1 regulates granule cell migration and cortical folding of the cerebellar cortex. Dev Biol. 2017;432(1):165-177. doi:10.1016/j.ydbio.2017.09.036<br>4. Men, Y., Zhang, A., Li, H. et al. LKB1 Regulates Cerebellar Development by Controlling Sonic Hedgehog-mediated Granule Cell Precursor Proliferation and Granule Cell Migration. Sci Rep 5, 16232 (2015). https://doi.org/10.1038/srep16232
Data exclusions	No data were excluded.
Replication	Experimental results reflect replication of a minimum of three independent trials comprised of multiple cohorts.<br>Fluorescence intensity measurements were randomly taken in a fixed pixel dimension across images within the proper region of interest (e.g. cerebellar cortex vs. external granule layer).
Randomization	Animals within each genotype were randomly assigned to control or treatment groups.
Blinding	Investigators were not blinded to animal group allocation or data collection, but data acquisition and analysis of behavioral studies were blinded to the investigators by having random ear-tag number assigned to each subject mouse at the time of the experiments.

Reporting for specific materials, systems and methods

We require information from authors about some types of materials, experimental systems and methods used in many studies. Here, indicate whether each material, system or method listed is relevant to your study. If you are not sure if a list item applies to your research, read the appropriate section before selecting a response.

Materials & experimental systems

n/a	Involved in the study
☐	☒ Antibodies
☒	☐ Eukaryotic cell lines
☒	☐ Palaeontology and archaeology
☐	☒ Animals and other organisms
☒	☐ Clinical data
☒	☐ Dual use research of concern

Methods

n/a	Involved in the study
☒	☐ ChIP-seq
☒	☐ Flow cytometry
☒	☐ MRI-based neuroimaging

Antibodies

Antibodies used

Primary antibodies used in this study (Western blotting, IHC-IF, IF, ICC) are as follows:

anti-RBM4 (ProteinTech, 11614-1-AP),
anti-Pax6 (Millipore, AB2237),
anti-BrdU (ABclonal, A1482),
anti-Ki67 (Abcam, ab15580),
anti-calbindin (Abcam, ab108404),
anti-NeuN (Abcam, ab177487),
anti-cleaved caspase-3 (5A1E, Cell Signaling Technology, 9664),
anti-BDNF (Abcam, ab108319),
anti-TrkB (Cell Signaling Technology, 4603),
anti-phospho-TrkB Tyr816 (Millipore, ABN1381),
anti-phospho-TrkB Tyr817 (Invitrogen, MA5-32207),
anti-GAPDH (ProteinTech, 60004-1-Ig),
FLAG® (Sigma-Aldrich, F1804),
β-actin (ProteinTech, 66009-1-Ig), and
anti-α-Tubulin (Millipore, 05-829).

Secondary antibodies used in this study are as follows:

Anti-Mouse IgG, HRP-conjugated (GE Healthcare, Cat# NA931)
Anti-Rabbit IgG, HRP-conjugated (GE Healthcare, Cat# NA934)
Goat anti-Mouse AlexaFluor™ Plus 488 (Thermo Fisher Scientific, Cat# A32723)
Goat anti-Rabbit AlexaFluor™ Plus 488 (Thermo Fisher Scientific, Cat# A32731)
Goat anti-Mouse AlexaFluor™ 568 (Thermo Fisher Scientific, Cat# A-11004)
Goat anti-Rabbit AlexaFluor™ 568 (Thermo Fisher Scientific, Cat# A-11011).

Validation

These antibodies were quality-checked by either manufacturers or previous publications.

Animals and other research organisms

Policy information about studies involving animals; ARRIVE guidelines recommended for reporting animal research, and Sex and Gender in Research

Laboratory animals

Mouse: EIIa-Cre; Rbm4dKO (C57BL/6J) and wildtype C57BL/6J (Jackson Laboratory).
Mice were maintained in a 12 h-dark/light cycle at specific pathogen free facility (Experimental Animal Facilities, Academia Sinica), with standard food and water provided ad libitum. Eight-week-old male mice were used for the behavioral tests. E18.5 to eleven-week-old mice were used for histological and biochemical analyses.

Wild animals

No wild animals were involved in this study.

Reporting on sex

The gender profile was recorded for behavioral studies, but there existed no gender effect in the behaviors within each genotype. Regarding primary cerebellar granule cell cultures, cerebella (5-9 per batch) isolated from both male and female pups at age of P7 were mixed to exclude any effect of biological sex, which was not considered in study design.

Field-collected samples

No field-collected samples were involved in this study.

Ethics oversight

All animal care and experimental protocols were reviewed and approved by the Institutional Animal Care and Use Committee (IACUC, protocol IDs 13-04-547 and 19-12-1370) of Academia Sinica and compliant with the Ministry of Science and Technology, Taiwan.

Note that full information on the approval of the study protocol must also be provided in the manuscript.
